# Telomere Attrition With Concomitant hTERT Overexpression Involved in the Progression of Gastric Cancer May Have Prognostic and Clinical Implications in High-Risk Population Group From North India

**DOI:** 10.3389/fonc.2022.919351

**Published:** 2022-07-13

**Authors:** Ifra Mushtaq, Gh Rasool Bhat, Bilal Rah, Syed Besina, Sheikh Zahoor, Muneer A. Wani, Mubashir A. Shah, Sadaf Bashir, Muzamil Farooq, Rafiq A. Rather, Dil Afroze

**Affiliations:** ^1^ Advanced Centre for Human Genetics, Sher-I- Kashmir Institute of Medical Sciences, Srinagar, India; ^2^ Department of Pathology, Sher-I- Kashmir Institute of Medical Sciences, Srinagar, India; ^3^ Department of Surgical Oncology, Sher-I- Kashmir Institute of Medical Sciences, Srinagar, India; ^4^ Department of General Surgery, Sher-I- Kashmir Institute of Medical Sciences, Srinagar, India

**Keywords:** gastric cancer (GC), c-Myc, human telomerase reverse transcriptase (hTERT), telomeres, North India

## Abstract

Genetic instabilities exacerbated by the dysfunction of telomeres can lead to the development of cancer. Nearly 90% of all human malignancies are linked with telomere dysregulation and overexpression of telomerase, an enzyme that catalyzes the synthesis of telomeric DNA repeats at the ends of chromosomes. The burden of gastric cancer continues to inflict a deterring impact on the global health scenario, accounting for over one million new cases in 2020. The disease is asymptomatic in its early stages of progression, which is attributed to the poor prognosis and overall surge in mortality rate worldwide. Exploiting telomere physiology can provide extensive mechanistic insight into telomere-associated gastric cancer progression and its use as a target in a variety of therapeutic interventions. In this study, we aimed to evaluate the clinical implications of c-Myc, human telomerase reverse transcriptase (hTERT) expression, and telomere length in patients with gastric cancer. A total of 57 gastric cancer cases and adjacent controls were included in the study. RT-PCR and immunohistochemistry were used to assess the expression levels of c-Myc and hTERT. The relative telomere length was measured by MMQPCR using the Cawthon method. Our results indicated that the shorter telomere and increased hTERT expression were associated with gastric cancer progression. The study also highlighted the role of short telomeres and increased expression of hTERT in gastric cancer progression and its association with various etiological risk factors, transcriptional activators, and overall survival among the ethnic Kashmiri population of North India.

## Introduction

Gastric cancer (GC) is a heterogeneous disease and asymptomatic in its early stages, which is attributed to its poor prognosis. The disease continues to have a deterring impact on global health, accounting for 768,793 deaths in 2020. It is one of the most prominently diagnosed cancers in men, where the incidence rates are twofold higher than in women (1). Genetic predisposition, epigenetic modifications, and the influence of various environmental factors, including *Helicobacter pylori* infection, smoking, alcohol consumption, and other dietary factors have, been associated with the pathogenesis of the disease (2). Despite the availability of various therapeutic modalities, the 5-year survival rate of patients diagnosed with gastric cancer remains disappointing (~10%–25% only) (3). In the current study, we tried to understand the regulatory mechanism of telomere in the disease pathogenesis and survival outcomes among gastric cancer patients, which might be helpful in exploring the various therapeutic targets and personalized treatment options for the early diagnosis and screening of high-risk groups in the genetically less explored Kashmiri population, North India.

Telomerase repression and maintenance of telomere length represent the key hallmarks of tumor suppressor pathways. Telomeres are the highly conserved, dynamic structures present at the chromosomal ends that maintain genomic stability by preventing the activation of DNA damage-causing mechanisms. Telomeres are composed of the double-stranded nucleotide repeats of 5′-TTAGGG-3′ that form a larger looped structure (T loop) upon binding with a complex of six proteins termed the Shelterin complex (4–6). The Shelterin complex maintains the telomere length and prevents the activation of inappropriate DNA damage response (DDR) pathways and nonhomologous end-joining (NHEJ) mechanisms (7). Telomerase is a ribonucleoprotein complex that includes a catalytic subunit—telomerase reverse transcriptase (TERT), an RNA template (telomerase RNA component (TERC)), and a complex of other associated proteins (8). Human telomerase reverse transcriptase (hTERT) is the only rate-limiting component of telomerase that plays a major role in process of immortalization and cellular aging (9). Transcriptional activation of hTERT is mainly regulated by various transcriptional activators and repressors. One of the positive regulators of hTERT is the oncogene c-Myc, which directly binds to the proximal region of the hTERT promoter in association with other transcriptional factor domains, thereby regulating its expression. Overexpression of c-Myc is implicated in many cancers, including GC, human fibroblasts, or epithelial cells where it directly targets hTERT to increases its expression and activity (10). Thus, understanding the molecular mechanisms underlying the hTERT regulation and its effect on telomere length will help us in understanding and accessing the risk and prognosis of GC in our Kashmiri population.

## Materials and Methods

### Specimen Collection

The current study was approved by the Institutional ethics committee of the Sher-i-Kashmir Institute of Medical Sciences (SKIMS) under notification no. **#**RP 06/2019. Tissue samples (GC cases and adjacent normal tissues) along with the peripheral blood were collected from 57 GC patients recruited from 2018 to 2021 from the Department of General and Minimal Invasive Surgery and the Department of Surgical Oncology, SKIMS.

Moreover, only the subjects who underwent surgical resection and were histopathologically confirmed to have GC without any history of other malignancy were included in the study. Proper information consent for clinical and family history was obtained prior to their surgeries. The patients were on frequent follow-ups after surgery to collect all the necessary information.

### DNA Extraction

Genomic DNA was extracted using a DNA extraction kit (HIMEDIA; MB504) and/or manually by the standard phenol/chloroform-isoamyl (PCI) method. The quality and quantity were determined at an absorbance of 260 and 280 nm using a spectrophotometer (Eppendorf Biospectrometer, Hamburg, Germany) or by 0.8% agarose gel electrophoresis. Pure DNA samples with an A_260_/A_280_ ratio of 1.8–2.0 were processed for the study.

### Telomere Length Assay

Relative telomere length was determined by the real-time quantitative PCR method using specific PCR primers for telomeres and single-copy genes. The reaction was performed under well-calibrated PCR conditions. The relative telomere length was measured as the T/S ratio (=2^−ΔΔct^), which represents the ratio of telomere repeat copy number to single gene copy number. The telomere primer sequence and single-copy gene *36b4f* primer sequence were as follows: *Tel* (forward) 5′-CGGTTTGTTTGGGTTTGGGTTTGGGTTTGGGTTTGGGTT-3′, *Tel* (reverse) 5′-GGCTTGCCTTACCCTTACCCTTACCCTTACCCTT-ACCCT-3′, and *36B4* (forward) 5′-CAGCAAGTGGGAAGGTGTAATCC-3′, (reverse) 5′-CCCATTCTATCATCAACGGGTACAA-3′. The reaction mixture was prepared using 10 µM primers in a 20-µl reaction using 2× SYBR Green qPCR Master Mix (Thermo Fisher Scientific). The DNA used was diluted to a final concentration of 10 ng/µl. The thermal profile conditions consisted of the following: an initial 10-min denaturation at 95°C, followed by 45 cycles of 95°C hold for 15 s, 60°C annealing for 30 s, and 72°C hold for 11 s.

### RNA Extraction

Total RNA was extracted from the blood/tissue samples using Trizol reagent (Ambion Life Technologies; #15596026). Quality and concentrations were quantified using a UV-spectrophotometer (Eppendorf Biospectrometer^®^, Hamburg, Germany) at an absorbance of A_260/280_. Briefly, 1.0 µg of RNA was reverse transcribed to cDNA using the Revertaid First-Strand cDNA Synthesis Kit (Thermo Fisher Scientific) in a final volume of 20 µl reaction containing 1× reverse transcriptase (RT) buffer, M-MLVRT (reverse transcriptase), RiboLock, RevertAid, and random hexamer. The reaction conditions for cDNA synthesis were as follows: 42°C hold for 60 min, 70°C hold for 5 min, and 4°C hold for infinite.

### Real-Time PCR for C-Myc and hTERT Expression

Quantitative real-time PCR was performed on Rotor-Gene (QIAGEN) detection systems with specific primers for: *c-Myc* (Forward: 5′-CCAGTAGCGACTGTGAAGGAAG-3′, Reverse: 5′-AGCTGGAGTAGTCGCTCTGC-3′), *hTERT* (Forward: 5′-TGAACTTGCGGAAGACAGTG-3′,Reverse: 5′-AGCTGGAGTAGTCGCTCTGC-3′) and *β-actin* (Forward: 5′-AGCGAGCATCCCCCAAAGTT-3′, Reverse: 5′-GGGCACGAAGGCTCATCATT-3′).

The reaction mixture contained PCR primer mix and Maxima SYBR Green qPCR Master Mix (2×). Corresponding Ct values and melting curves were obtained, and the relative gene expression was calculated. The reaction profile consisted of 45 cycles with an initial denaturation of 95°C for 10 min, a hold of 95°C for 15 s, annealing of 58°C for 30 s, and a final hold of 72°C for 10 s. The mRNA expression was measured by the 2^−ΔΔct^ method (Livak Method).

### Immunohistochemical Staining and Quantification

Immunohistochemistry was performed on 5-μm-thick sections of formalin-fixed, paraffin-embedded (FFPE) tissue samples which were prepared on poly-l-lysine-coated slides. The slides were subjected to antigen retrieval using citrate buffer (pH 6.0) in an autoclave. The primary antibodies hTERT (GeneTex GTX30410) and c-Myc (GeneTex GTX103436) were used for overnight incubation of sections at 4°C. After washing three times with washing buffer (PBS), the slides were treated with secondary antibody (Anti-Polyvalent HRP Polymer, Jackson ImmunoResearch). Detection was done using diaminobenzidine as chromogen according to the manufacturer’s protocol. Immunohistochemical (IHC) quantification was done by semiquantitative H-score (stained intensity and positive cell percentage). The staining intensity was graded as 0 for no staining; 1+ (10%–50%) for weak staining, 2+ (50%–70%) for moderate staining; and 3+ (>70%) for intense staining.

### Statistical Analysis

All statistical analyses were performed using Graph-Pad prism version 8.0 software. The Chi-square test and Student’s *t*-tests were used to analyze different variables as applicable, and data were presented as mean ± SEM as appropriate. To estimate the proportion surviving at a point in time and to compare the overall survival curves from different groups, the log-rank test and Kaplan–Meier curve were used.

## Results

A total of 57 GC patients recruited in the study showed a mean age of ±59.91 years with a male preponderance (*n* = 41) to females (*n* = 16). The various clinicopathological characteristics of the patients are listed in **Table 1**.

**Table 1:** The various characteristic features of gastric cancer patients.

Clinicopathological parameters	Controls	Cases	p-value
***Age***			
***≥51***	22 (55%)	44 (77%)	**0.02**
**≤50**	18 (45%)	13 (28%)	
**Gender**			
**Males**	30 (75%)	41 (71.9%)	0.37
**Females**	10 (25%)	16 (28.07%)	
**Smoking status**			
**Yes**	4 (10%)	33 (57%)	**<0.01**
**No**	36 (90%)	24 (42.1%)	
***Histopathology***			
**Poorly differentiated adenocarcinoma**	–	23 (40.35%)	
**Moderately differentiated adenocarcinoma**	–	20 (35.08%)	
**Well-differentiated adenocarcinoma**	–	7 (12.28%)	
**Others**	–	7 (12.28%)	
***TNM staging***			
**Stage I**	–	5 (8.7%)	
**Stage II**	–	19 (33.33%)	
**Stage III**	–	33 (57.89%)	
**Life style**			
**Active**	32 (80%)	50 (87.7%)	0.27
**Sedentary**	8 (20%)	7 (12.28%)	
**Demography**			
**Rural**	15 (37.5%)	47 (82.45%)	**<0.01**
**Urban**	25 (62.5%)	10 (17.54%)	

The bold values represents that they are statistically significant.

### Increased Expression of C-Myc Is Associated With GC Progression

**Figure 1:**
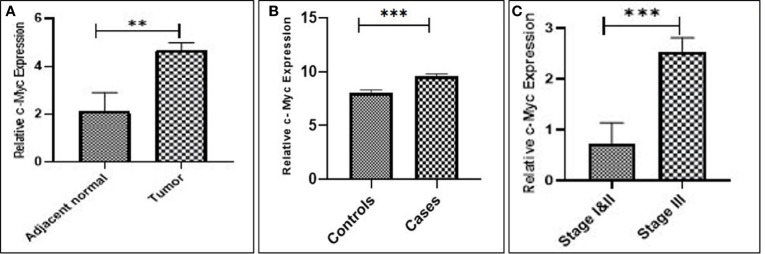
Upregulation of c-Myc expression in gastric cancer. **(A)** Tissue. **(B)** Blood. **(C)** Stage III compared to stages I and II.

Real-time expression profiling results revealed that c-Myc expression was significantly higher in 44 of 57 (77.1%) cases as compared to their respective controls in both tissues and blood samples (*p* = 0.006; 0.0003), respectively. No statistically significant difference in c-Myc expression was observed in terms of gender, age, smoking status, and histological classification. However, elevated expression of c-Myc was observed in the advanced stage with profound tumor invasion and lymph node involvement, as compared to the early stages of GC (*p* = 0.0009), as shown in **Figure 1** and **Table 2**.

**Table 2:** Association of c-Myc, hTERT expression, and relative telomere length with different clinicopathological findings in gastric cancer patients.

Clinicopathological parameters	c-Myc expression			hTERT expression			Relative telomere length		
	Low expression	High expression	p-value	Low expression	High expression	p-value	Short telomeres	Long telomeres	p-value
**Gender**									
**Males**	08	33	**0.76**	12	29	**0.3**	32	10	**0.006**
**Females**	06	10		07	09		08	07	
**Age**									
***≥51***	12	32	**0.89**	14	30	**0.03**	32	12	**0.002**
**≤50**	01	12		5	8		08	05	
**Smoking status**									
**Smokers**	07	26	**0.3**	10	23	**0.58**	26	07	**0.02**
**Nonsmokers**	07	17		09	15		14	10	
**Histopathology**									
**Poorly differentiated**	05	18	**0.18**	09	14	**0.67**	16	07	**0.8**
**Moderately differentiated**	06	14		07	13		13	07	
**Well differentiated**	01	06		02	05		05	02	
**Others**	02	05		01	06		06	01	
**TNM staging**									
**Stages I and II**	07	14	**0.0009**	09	15	**0.01**	12	09	**0.4**
**Stage III**	07	29		10	23		28	08	

The bold values represents that they are statistically significant.

### Upregulation of hTERT Is Associated With GC Progression

**Figure 2:**
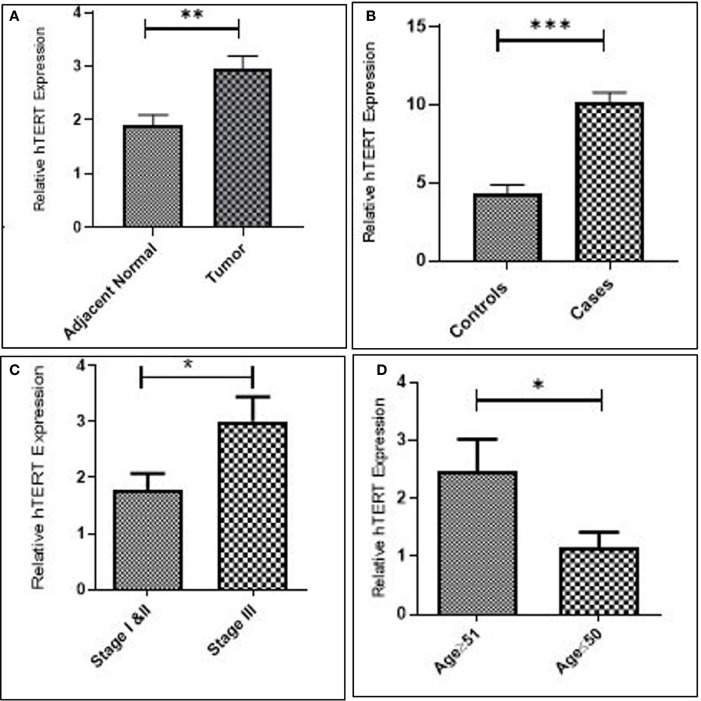
Increased expression of hTERT in gastric cancer. **(A)** Tissue. **(B)** Blood. **(C)** Late stage. and **(D)** Advanced age.

Increased expression of hTERT was observed in both GC tissues (*p* = 0.0054) and blood samples (*p* = 0.0009) as compared to their corresponding controls. Additionally, upregulated expression of hTERT was significantly associated with advanced tumor node metastasis (TNM) staging (*p* = 0.01), and interestingly, increased age of GC patients was significantly associated (*p* = 0.03), as in **Figure 2** and **Table 2**.

### Immunohistochemistry of C-Myc and hTERT in GC Patients

**Figure 3:**
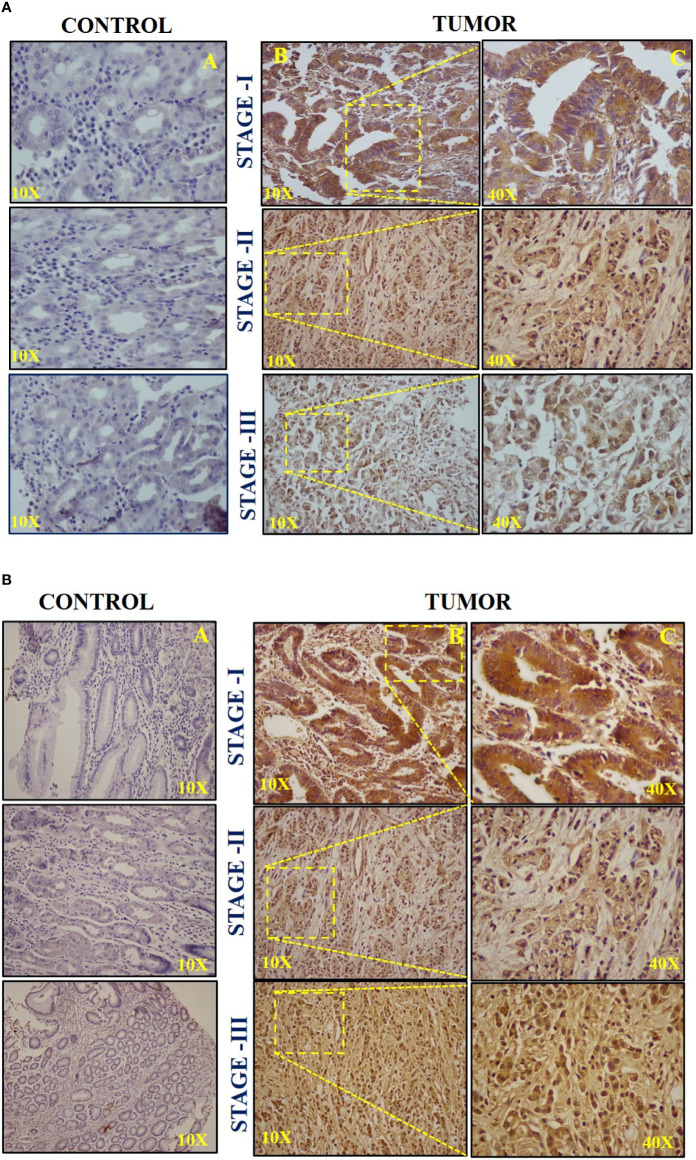
**(A)** Representative images of immunohistochemistry (IHC) of c-Myc in (A) stage I, (B) stage II, and (C) stage III. **(B)** Representative images of immunohistochemistry (IHC) of hTERT in (A) stage I, (B) stage II, and (C) stage III.

The patients were categorized based on different histological grades and TNM staging. In total, 23 (40.35%) of 57 patients had poorly differentiated adenocarcinoma, 20 (35.08%) had moderately differentiated adenocarcinoma, 7 (12.28%) belonged to well-differentiated adenocarcinoma, whereas 7 (12.28%) had other distinct types of gastric carcinoma. To further elucidate the expression and localization of c-Myc and hTERT, immunohistochemistry was performed in 57 GC tissues. Out of 57 cases, high expression of c-Myc was observed in 45 (80%) of them, whereas 37 (65%) showed high expression of hTERT. Samples positive for c-Myc showed high expression in the nucleus; however, in some of the cases, cytoplasmic staining was also detected, as in **Figure 3A**.

High hTERT expression was seen in both the nucleus and cytoplasm. The H-score of +++ was considered for intense staining, ++ for moderate staining, and + for weak staining. Stage III displayed a high H-score (++/+++) for both c-Myc and hTERT compared to stages I and II, respectively, as shown in **Figure 3B**. Moreover, the IHC scoring result for both c-Myc and hTERT is shown in **Supplementary Figures 1A, B**. Furthermore, it was observed that high H-score in stage 3 was statistically significant when compared to stages 1 and 2 in c-Myc (*p* = 0.0184) and hTERT (*p* = 0.008), respectively.

### Relative Telomere Length and Its Association With Clinicopathological Features in GC

The overall telomere length measured by real-time PCR was found to be relatively shorter in GC cases compared to healthy controls (*p* = 0.0002). Since GC is a late-onset disease that progresses slowly, the enrolled patients were segregated into two age groups (age group ≥51 and age group ≤50). Telomere length was inversely associated with age and was found to be considerably shorter among the age group ≥50 (*p* = 0.002). Moreover, the relative telomere length was significantly shorter among men than women (*p* = 0.006) in the ethnic Kashmiri population of North India.

**Figure 4:**
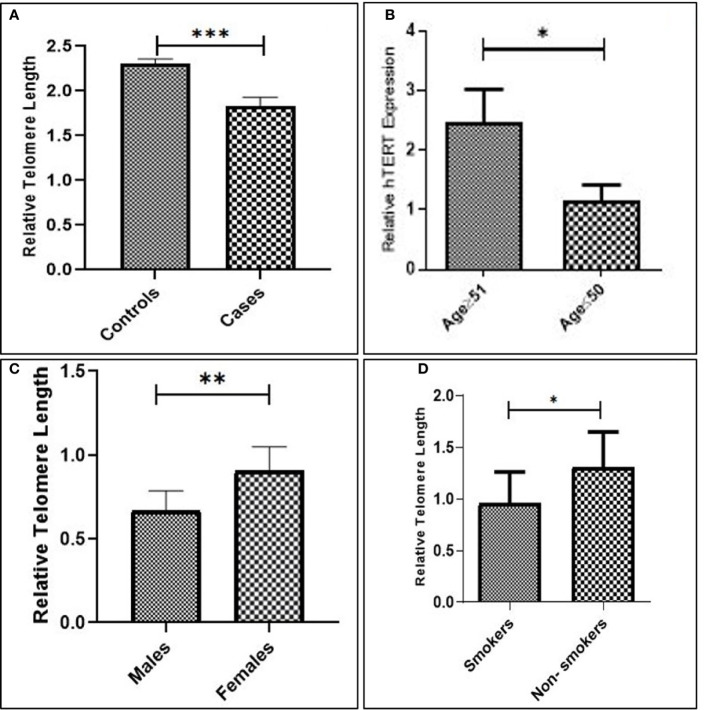
**(A)** Relative telomere length among cases and controls. **(B)** Advanced age. **(C)** Males compared to females. **(D)** Smokers compared to nonsmokers.

Since smoking is considered to be one of the contributing factors responsible for the etiology of the GC, we intended to correlate telomere length with the smoking status among the study groups. The relative telomere length was found to be significantly shorter among smokers as compared to nonsmokers (*p* = 0.02), as shown in **Figure 4** and **Table 2**.

### Association of C-Myc, hTERT Expression, and Telomere Length With Overall Survival in GC Patients

**Figure 5:**
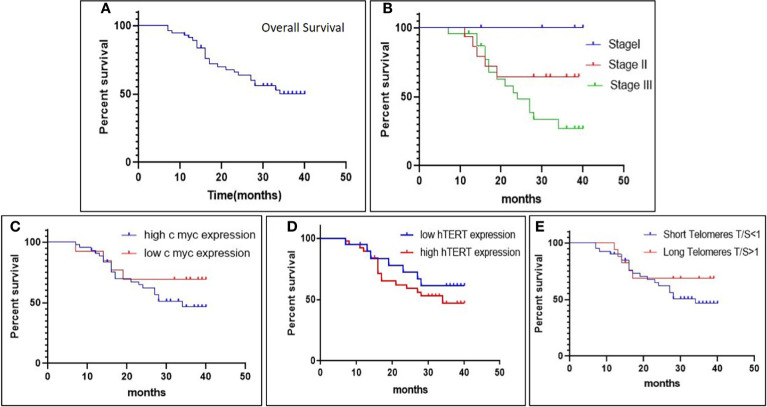
The Kaplan–Meier survival plot depicts **(A)** overall survival (OS) in gastric cancer patients; **(B)** overall survival (OS) in different stages; **(C, D)** association of overall survival (OS) with c-Myc expression and hTERT expression; and **(E)** association of overall survival (OS) with relative telomere length.

Kaplan–Meier survivor functions predicted that approximately 56.14% of our study groups are censored, and over a period of 40 months, 43.85% of the patients have died. The overall survival (OS) of late-stage groups as depicted by the log-rank test was worse than that of early-stage groups (*p* = 0.01). Increased expression of c-Myc was associated with overall poor prognosis (*p* = 0.2) (hazard’s ratio = 1.685; CI = 0.7267–4.457). Similarly, high hTERT expression was associated with poorer survival than groups with low hTERT expression (*p* = 0.3) (hazard’s ratio = 1.452; CI = 0.6352–3.319). Thus, our results revealed that the poor prognosis of GC can be attributed to the presence of high c-Myc and hTERT expression. Short telomeres showed worse overall survival (OS) with a greater number of deaths as compared to patients with long telomeres (*p* = 0.3) (hazard’s ratio = 1.594; CI = 0.6964–3.964), as in **Figure 5**.

**Figure 6:**
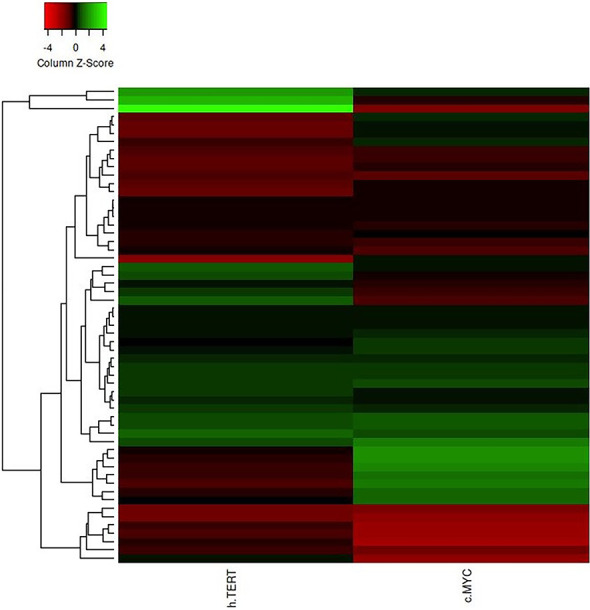
The heat map plot of hTERT and c-Myc expression. Columns of the plot represent the genes and the rows represent samples, respectively.

**Figure 7:**
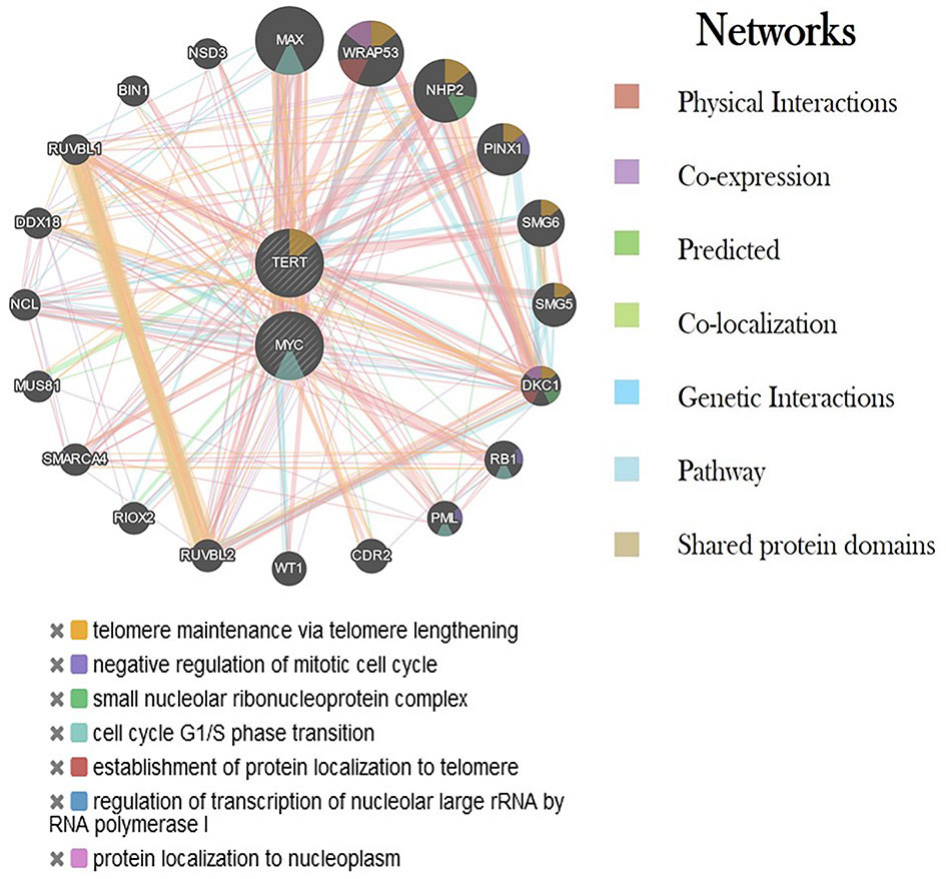
Interaction of hTERT and c-Myc genes with other critical genes using various networks. Each color represents a different type of interaction and its role in various biological processes.

The differential expression of the hTERT and c-Myc was also represented by heat map plot analysis. As shown in **Figure 6**, the columns of the plot represent the genes and the rows represent the samples, respectively. Moreover, the gene–gene interaction analysis of hTERT and c-Myc revealed that *MAX*, *NHP2*, *DKC1*, *RUVBL2*, and *RUVBL2* showed maximum interaction as in **Figure 7**. These genes might play a role in GC progression and could be explored for future studies.

## Discussion

Telomeres are the hexanucleotide repeats of the TTAGGG sequence, present at the distal ends of human chromosomes, and are important for chromosome integrity and genomic stability. Every round of genome replication has the possibility of causing genetic abnormalities and chromosomal changes that can drive the cell towards the earlier process of oncogenesis (11, 12). Telomerase is a ribonucleoprotein that synthesizes telomeric DNA using an RNA template. Cells that have the active enzyme appear to be immune to telomeric shortening with endless potential for growth (13, 14). Multiple tumor cell lines have been shown to have high hTERT expression and telomerase activity, which is undetectable in normal tissues.

In the present study, we highlighted the role of telomere length as assessed by real-time PCR. The findings of our study revealed that telomere attrition was significantly associated with the higher incidence of GC, and the association was also consistent with other clinicopathological parameters as well. The majority of early research also linked short telomeres to an increased risk of GC (15, 16). The relative telomere length was found to be shorter in GC patients when compared to healthy controls. When the telomere length was correlated with gender, it was found to be significantly shorter among men than in women. This discrepancy might be due to the fact that the incidence of GC is twofold higher in men compared to women and due to the small population subset of female groups in our study. This is in line with the previous studies showing that the length of the telomere was substantially shorter in GC patients than in their normal counterparts (17). When the study subjects were segregated into two age groups with age ≥51 and age ≤50, short telomere length was significantly associated among the age group ≥51. These finding further ascertains the late-stage diagnosis of GC. The underlying cause of a stronger relationship in younger people is unknown. While some of the studies have identified a correlation between short telomeres and an increased risk of GC in both young and old subjects (18), a few of the studies have found a causal relationship in older people only (16). The association between telomere length and the GC risk could be explained by the fact that short telomeres can cause chromosomal instability, which can lead to carcinogenesis, whereas long telomeres can promote cell division and increase the probability of aberrations, which can lead to the development of cancer (19).

Smoking is one of the most significant environmental risk factors for developing GC. Studies have shown that smokers are at a greater risk of predisposition to GC. Moreover, studies have also reported the association of short telomere lengths with smoking status (20). We intended to correlate telomere length with the smoking status among the cases. The relative telomere length was found to be significantly shorter among the smokers when compared to the nonsmoker subjects.

Moreover, Kaplan-Meier survivor functions were plotted for GC patients based on their relative telomere lengths, it was noted that the shorter telomere length is associated with a poor prognosis of GC. The shortening of telomeres leads to cancer susceptibility due to genomic instability. The association of short telomeres with the disease survival among GC patients has previously been reported (21).

**Figure 8:**
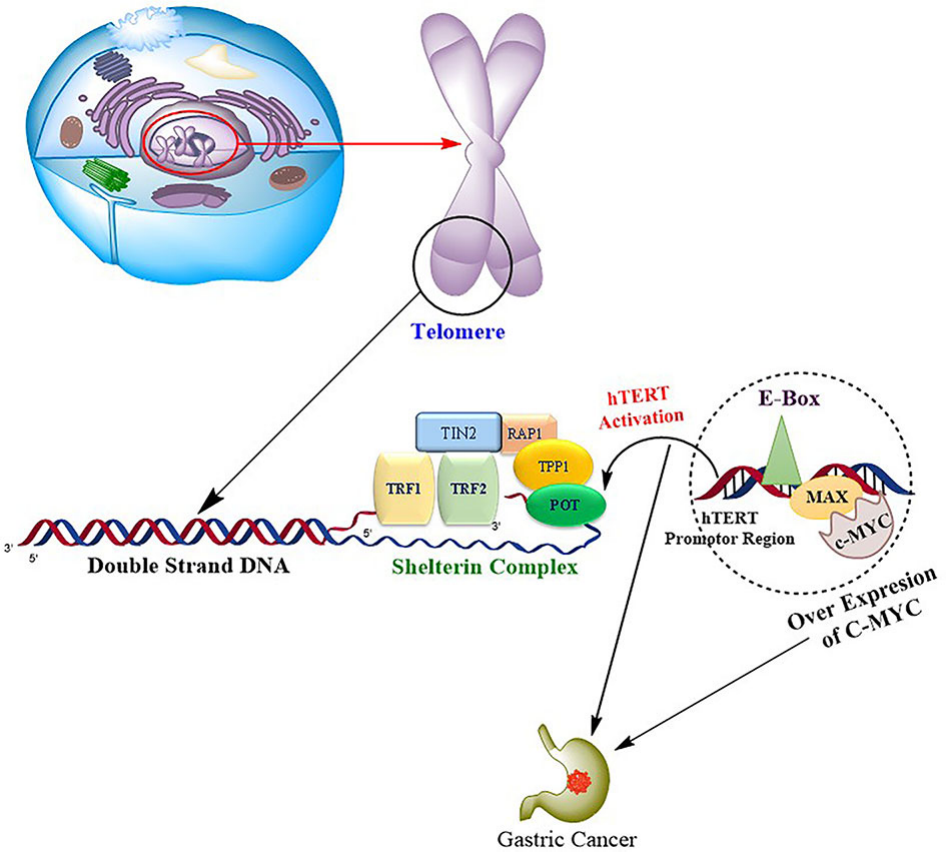
Progression of gastric carcinoma is mediated by the binding of oncogene c-Myc to the hTERT promoter region *via* transcription factor domains MAD/MAX complex, resulting in upregulation of hTERT, which in turn causes the repeated synthesis of telomeric DNA and drives the cell toward tumorigenesis.

Due to hTERT transcriptional suppression, somatic cells lose telomerase activity, resulting in telomere shortening and eventually cellular senescence. hTERT expression and subsequent telomerase activation are critical for telomere length stabilization and are thus required for malignant cells to overcome mechanistic senescence checkpoints and acquire the ability to proliferate indefinitely. In several types of human cancers, including GC, hTERT monitoring has been shown to be a valuable diagnostic and prognostic marker (22). Numerous studies have been published to date that show the significance of hTERT in tumor invasion and metastasis in a variety of tumor types such as melanoma, glioma, and GC, hepatocellular, but only a few have studied the mechanism behind this coherence (23–28). Regulation of hTERT is attained through various transcriptional factors like c-Myc, Sp1, STAT3, and NF-κB. c-Myc represents one of the important regulators that modulate hTERT transcription directly or indirectly under the influence of other signaling pathways and transcription factors (29). c-Myc is an oncoprotein that is dysregulated in various oncogenic processes where its targets are involved in cellular proliferation, differentiation, and apoptosis. The presence of two E-boxes (5′-CACGTG-3′) at −165 and +44 on the TERT core promoter creates a binding site for several transcriptional factors. c-Myc forms a heterodimer with one of the transcriptional factor domains Max and binds to the E-boxes in the core promoter region of hTERT to activate its transcription (30). c-Myc also acts as the central downstream target for various signaling pathways like NF-κB. Upregulation of this pathway results in the activation of c-Myc, which in turn leads to the indirect activation of hTERT (31). A study by Silva et al. (32) reported that increased c-Myc and hTERT expression occurs simultaneously in GC, suggesting a positive correlation between the two genes, as shown in **Figure 8**. Increased expression of c-Myc and its association with poor prognosis of GC has been previously studied (33). Our results are consistent with earlier studies of increased expression of c-Myc and its association with the disease progression, deep invasion, and poor survival outcomes.

We also evaluated the mRNA expression levels of hTERT in both gastric cancer tissue and blood. High expression of hTERT was observed in gastric carcinoma tumor tissue when compared to the corresponding adjacent tumor counterpart and blood samples of the GC patients. These results are consistent with the previous investigations, which also revealed the increased hTERT mRNA expression in GC (34). Consistent with the previous findings, increased hTERT expression was associated with the late stages of the disease and with the poor survival of patients.

In conclusion, our study indicated a putative biological process that demonstrates that activation of c-Myc caused by various genetic mutations leads to the consequent activation of hTERT expression in GC. In our population, short telomeres are associated with an increased risk of GC and a poor prognosis. Overall, our study was successful in addressing the association of c-Myc, hTERT, and telomere length with various clinicopathological parameters in our patients. Together, these findings may pave the way for the development of various targets and their use as therapeutics for the early detection and improvement of the overall survival of GC patients. The small sample size is the limitation of the present study.

## Data Availability Statement

The original contributions presented in the study are included in the article/**Supplementary Material**, further inquiries can be directed to the corresponding author/s.

## Ethics Statement

The studies involving human participants were reviewed and approved by Institutional Ethics Committee (IEC-SKIMS) #RP 06/2019. The patients/participants provided their written informed consent to participate in this study.

## Author Contributions

IM and DA conceived the concept. IM, GB, and DA performed the data analysis. SB, SZ, MW, and MS provided the clinical samples. BR, SB, MF, and RR helped in the technical refinement of the manuscript. All authors listed have made a substantial, direct, and intellectual contribution to the work and approved it for publication.

## Funding

We acknowledge DST-SERB, Department of Science and Technology, New Delhi, India (EMR/2016/004794) and an Institutional Intramural grant (SKIMS, Soura #RP 06/2019) for the accomplishment of this study.

## Conflict of Interest

The authors declare that the research was conducted in the absence of any commercial or financial relationships that could be construed as a potential conflict of interest.

## Publisher’s Note

All claims expressed in this article are solely those of the authors and do not necessarily represent those of their affiliated organizations, or those of the publisher, the editors and the reviewers. Any product that may be evaluated in this article, or claim that may be made by its manufacturer, is not guaranteed or endorsed by the publisher.
