# Supplementary figures and images for: Telomere Attrition With Concomitant hTERT Overexpression Involved in the Progression of Gastric Cancer May Have Prognostic and Clinical Implications in High-Risk Population Group From North India
Source: Front Oncol. 2022 Jul 13;12:919351. doi: 10.3389/fonc.2022.919351 (PMC9326504; doi:10.3389/fonc.2022.919351)

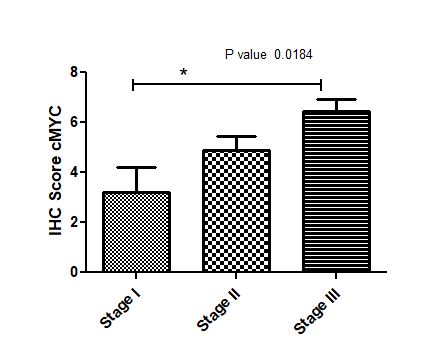

Supplement: Supplementary file 1 [file Image_1.jpg]

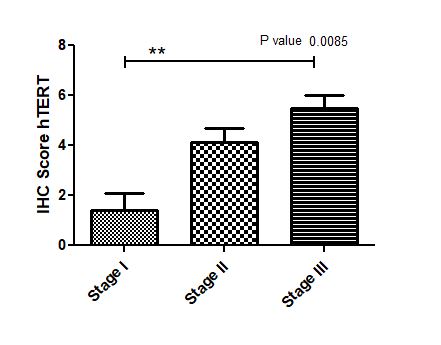

Supplement: Supplementary file 2 [file Image_2.jpg]
